# 

**Published:** 1847-10

**Authors:** 


CONTENTS

OF

### DENTAL REGISTER OF THE WEST.

### OCTOBER NO., 1847.

Art. 1, Proceedings of the Mississippi Valley Association of Dental Surgeons, Third Annual Meeting,........ Page 1
Art. 2, Address by the President, Dr. E. Taylor,........ 5
Art. 3, Address on Tobacco, by A. Berry, D. D. S.,........ 12
Art. 4, Galvanic Action and Amalgam, by E. Slack, M. D.,........ 18
Art. 5. Contour of the Face, by J. Allen, D. D. S.,........ 24
Art. 6, Destroying Nerves of the Human Teeth, by J. Allen, D. D. S.,. 25
Art. 7, On Filling Teeth, by E. Taylor, M. D., D. D. S.,........ 27
Art. 8, Diseased Gums, by H. R. Smith, D. D. S.,........ 30
Art. 9, Nerve Killing, by M. Rogers, M. D., &c.,........ 34
Art. 10, Third Dentition, by J. Sanford, Dentist,........ 39
Art. 11, Letheon,........ 40
Art. 12, Use of the Letheon, by Joseph Taylor, D. D. S.,........ 41
Art. 13, Rules for the Exhibition of the Letheon,........ 44
Art. 14, Letter from H. Crane, D. D. S.,........ 45
Art. 15, Anecdote " " ........ 46
Art. 16, Letter from S. P. Hullihen, M. D.,........ 47
Art. 17, Tooth Ache, R. C. Carter, M. D.,........ 48
Art. 18, Staple Rivet Teeth,........ 48
Art. 19, The Latest Fashion on the New Plan,........ 50
Art. 20, The First No. of the Register,........ 51
Art. 21, The Second No. " ........ 52
Art. 22, Ohio College of Dental Surgery,........ 53
Art. 23, Popular Prejudices in relation to the Decay of the Teeth,...... 53

